# B Cells in Patients With Melanoma: Implications for Treatment With Checkpoint Inhibitor Antibodies

**DOI:** 10.3389/fimmu.2020.622442

**Published:** 2021-01-25

**Authors:** Zena N. Willsmore, Robert J. Harris, Silvia Crescioli, Khuluud Hussein, Helen Kakkassery, Deepika Thapa, Anthony Cheung, Jitesh Chauhan, Heather J. Bax, Alicia Chenoweth, Roman Laddach, Gabriel Osborn, Alexa McCraw, Ricarda M. Hoffmann, Mano Nakamura, Jenny L. Geh, Alastair MacKenzie-Ross, Ciaran Healy, Sophia Tsoka, James F. Spicer, Sophie Papa, Linda Barber, Katie E. Lacy, Sophia N. Karagiannis

**Affiliations:** ^1^ St. John’s Institute of Dermatology, School of Basic & Medical Biosciences, King’s College London, Tower Wing, Guy’s Hospital, London, United Kingdom; ^2^ Breast Cancer Now Research Unit, School of Cancer & Pharmaceutical Sciences, King’s College London, Guy’s Cancer Centre, London, United Kingdom; ^3^ School of Cancer & Pharmaceutical Sciences, King’s College London, Guy’s Hospital, London, United Kingdom; ^4^ Department of Informatics, Faculty of Natural and Mathematical Sciences, King’s College London, London, United Kingdom; ^5^ Department of Plastic Surgery at Guy’s, King’s, and St. Thomas’ Hospitals, London, United Kingdom; ^6^ Department of Medical Oncology, Guy’s and St Thomas’ NHS Foundation Trust, London, United Kingdom; ^7^ ImmunoEngineering, School of Cancer and Pharmaceutical Sciences, Faculty of Life Sciences and Medicine, King’s College London, London, United Kingdom

**Keywords:** melanoma, B cell, checkpoint inhibition therapy, antibody, humoral immune response

## Abstract

The contributions of the humoral immune response to melanoma are now widely recognized, with reports of positive prognostic value ascribed to tumor-infiltrating B cells (TIL-B) and increasing evidence of B cells as key predictors of patient response to treatment. There are disparate views as to the pro- and anti-tumor roles of B cells. B cells appear to play an integral role in forming tumor-associated tertiary lymphoid structures (TLSs) which can further modulate T cell activation. Expressed antibodies may distinctly influence tumor regulation in the tumor microenvironment, with some isotypes associated with strong anti-tumor immune response and others with progressive disease. Recently, B cells have been evaluated in the context of cancer immunotherapy. Checkpoint inhibitors (CPIs), targeting T cell effector functions, have revolutionized the management of melanoma for many patients; however, there remains a need to accurately predict treatment responders. Increasing evidence suggests that B cells may not be simple bystanders to CPI immunotherapy. Mature and differentiated B cell phenotypes are key positive correlates of CPI response. Recent evidence also points to an enrichment in activatory B cell phenotypes, and the contribution of B cells to TLS formation may facilitate induction of T cell phenotypes required for response to CPI. Contrastingly, specific B cell subsets often correlate with immune-related adverse events (irAEs) in CPI. With increased appreciation of the multifaceted role of B cell immunity, novel therapeutic strategies and biomarkers can be explored and translated into the clinic to optimize CPI immunotherapy in melanoma.

## Introduction

During early stages, primary melanoma lesions are removable through surgical intervention that is largely curative. In advanced disease however, melanoma can spread to regional lymph nodes and metastasize to distant sites. Historically, treatment options were limited in advanced disease to palliative cytotoxic chemotherapy, leading to poor 5-year survival rates. Over the last decade, immunotherapy, driven by checkpoint inhibitor antibodies, has transformed patient prognosis.

The rationale behind the use of checkpoint inhibitor therapy lies in the highly immunogenic nature of melanoma (1, 2). Melanoma carries a large mutational load, providing a range of tumor-specific antigens that are thought to elicit a host immune response. However, melanoma cells can evade immunosurveillance *via* activation of different immune-inhibitory pathways, including *via* immune checkpoint molecules and their downstream signals. Physiologically, checkpoint pathways play a role in immune homeostasis, providing negative feedback stimuli to prevent autoimmune reactivity. The best-studied checkpoint pathways are those of negative regulatory molecules cytotoxic T lymphocyte associated protein-4 (CTLA-4) and of the Programmed Death Receptor 1 (PD-1) and its ligands PD-L1 and PD-L2.

Checkpoint inhibitors (CPIs) were designed to promote immune-mediated elimination of tumor cells through modulation of T cell responses. Anti-CLTA-4 (Ipilimumab approved in 2011) (3) or anti-PD-1 (Pembrolizumab and Nivolumab approved in 2014) (3, 4) antibodies inhibit binding of checkpoint molecules with their respective ligand in the tumor microenvironment or draining lymph nodes. A newer anti-PD-L1 agent, Atezolizumab, was approved by the FDA in July 2020 (5). CPIs are approved for use as monotherapy in metastatic melanoma and as adjuvant therapy. More recently combination Nivolumab and Ipilimumab has been approved for metastatic disease in select patient groups. Their clinical efficacy and long-term outcomes are highlighted in the CheckMate 067 clinical trial (ClinicalTrials.gov NCT01844505) (4). In 945 patients with stage III or IV melanoma, this trial directly evaluated combination therapy with Nivolumab plus Ipilimumab in comparison with monotherapy with Nivolumab or with Ipilimumab. At a minimum follow-up of 60 months, the median overall survival (OS) was more than 60.0 months (median not reached) in the Nivolumab-plus-Ipilimumab group, 36.9 months in the Nivolumab group and 19.9 months in the Ipilimumab group. The 5-year OS rates were 52, 44, and 26% respectively (4).

Despite the undeniable clinical success of CPI therapy several challenges remain. There is currently a lack of biomarkers, limiting our ability to predict who will respond to treatment. Another important consideration is recognizing who will develop toxicities that are unfortunately common particularly with anti-CTLA-4 treatment and in the context of combination anti-CTLA-4 and anti-PD-1 therapy as compared with monotherapy. These toxicities, known as immune related adverse events (irAEs) vary in severity, can affect any organ system but most commonly target the skin, the gastrointestinal and the endocrine systems (6). Although many of these toxicities are manageable/reversible, they frequently lead to treatment disruption or discontinuation. Therefore, understanding the mechanisms underlying irAEs and predicting these irAEs is a much-needed clinical strategy.

**Figure 1:**
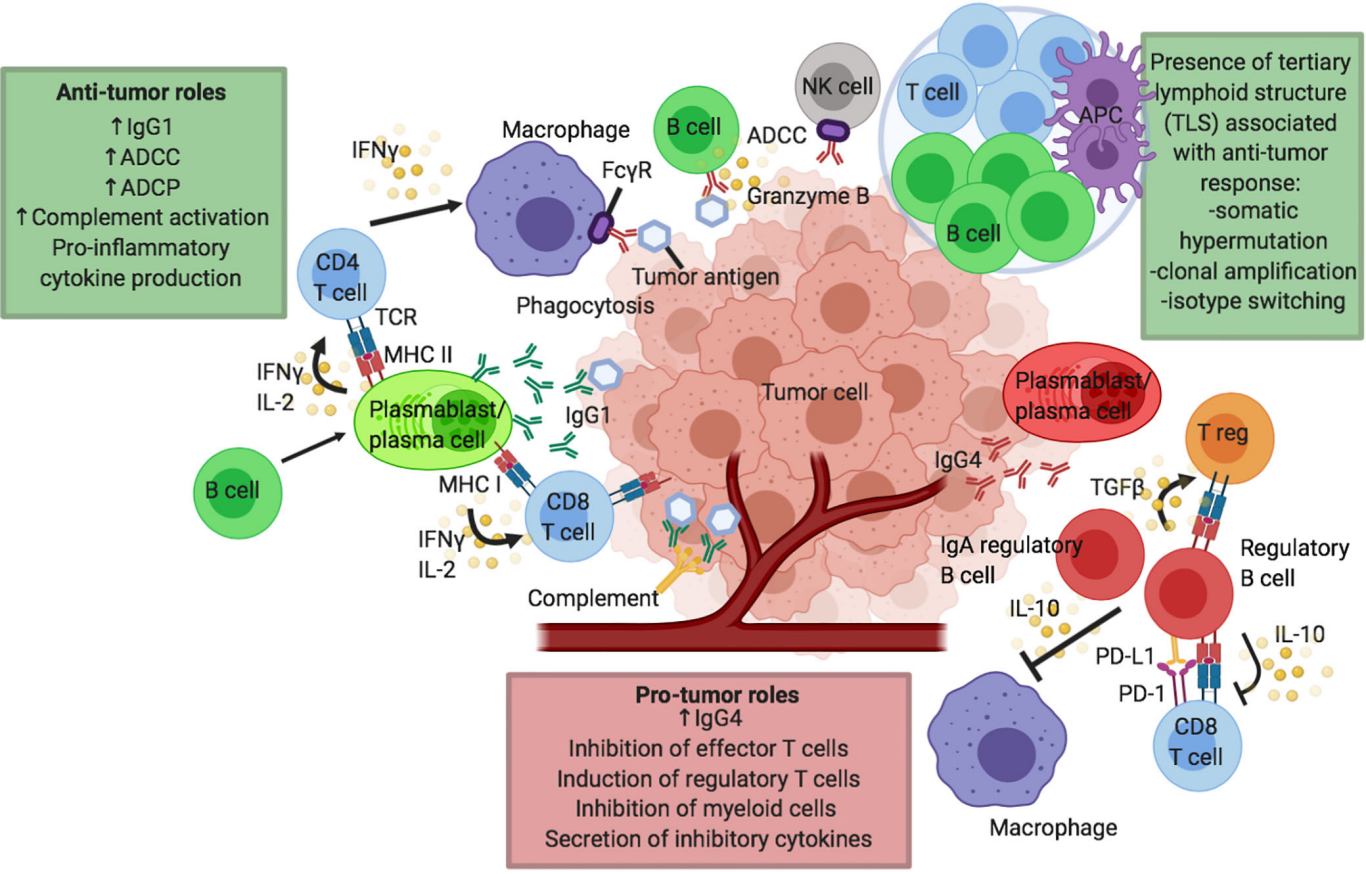
Proposed humoral responses in cancer and in response to checkpoint inhibitor immunotherapy. Putative pro- and anti-tumor roles of B cells in the tumor microenvironment (TME) and tumor tertiary lymphoid structures (TLS). B cells may confer several anti-tumor roles through production of effective tumor-clearing antibodies, predominantly IgG1: mediating antibody dependent cell-mediated cytotoxicity (ADCC), antibody dependent cell-mediated phagocytosis (ADCP) and facilitating complement activation. B cells can also effectively modulate CD4 and CD8 T cells *via* their functions as antigen presenting cells (APCs) to present tumor-derived antigens or by promoting cytokine secretion, particularly IFN*γ* and IL-2 by T cells. B cells can also support the presentation of tumor-derived antigens by other antigen presenting cells including macrophages and dendritic cells. Antigen presentation and B cell activation may be enhanced in TLS, leading to somatic hypermutation, clonal amplification and isotype switching to activatory antibody isotypes such as IgG1, further driving anti-tumor responses. B cells can also directly exert cytotoxic functions against tumor cells (*e.g.* through secretion of granzyme B). B cells can have pro-tumor roles by multiple mechanisms, for instance through expression of inflammatory antibody isotypes such as IgG4. IL-10 producing regulatory B cell (Breg) phenotype is described which can negatively modulate the function of effector CD8 T cells, induce the development of regulatory T cells (Tregs) and dampen the activity of antigen-presenting cells such as dendritic cells and macrophages. IgA producing regulatory B cells has also been described in the TME.

The role of T cells in CPI therapy has been extensively reviewed in literature. Contrastingly, B cell immunity has been less studied. B cells have a wide range of roles, including critical functions as professional antigen presenting cells (APCs), and they are capable of secreting cytokines and antibodies which enable them to conduct antibody-dependent cell-mediated cytotoxicity (ADCC), antibody-dependent cellular phagocytosis (ADCP) and complement-dependent cytotoxicity (CDC). Considering their comprehensive functions and with B cells making up one of the two arms of the adaptive immune system, it is not surprising to envisage a role within melanoma immunity and response to CPI. However, the exact role B cells play in tumor immunity is unclear with the existence of different cell subsets with juxtaposing functions, such as activatory and regulatory B cells (Bregs), and the presence of B cells in tertiary lymphoid structures (TLSs). The potential pro- and anti-tumor roles of B cells in melanoma are summarized in **Figure 1**. In this review we discuss emerging evidence for the significance of B cell and antibody immune responses in melanoma and specifically in the context of CPI therapy.

## The CTLA-4 and PD-1 Pathways

CTLA-4 is an inhibitor of T cell activation primarily expressed on naïve T cells after activation (7–10) and constitutively expressed on FoxP3+ regulatory T cells (Tregs) (10). T cell activation is dependent not only on T cell receptor (TCR) binding with an antigen presented *via* an APC, but also on the presence of a costimulatory second signal, typically through binding of CD28 on the T cell to CD80/86 on the APC. CTLA-4 is a competitive CD28 homolog that has a higher affinity to CD80 (B7-1) and to a lesser degree CD86 (B7-2) than CD28 therefore inhibiting T cell co-stimulation (10). TCR signaling up-regulates cell surface CTLA-4 expression, reaching peak expression at 2 to 3 days after activation (7, 8). This provides a negative feedback loop for T cell activation.

PD-1 is also a member of the immunoglobulin superfamily known to contribute to immune homeostatic processes by delivering inhibitory signals upon engagement with its ligands, programmed death ligands 1 and 2 (PD-L1/2). Like CTLA-4, PD-1 is thought to be a negative regulator of T cell function, regulating peripheral tolerance and T cell responses. PD-1 is expressed more broadly than CTLA-4 and can be found on T cells, B cells, natural killer (NK) cells, and a variety of peripheral tissues (11). Expression of PD-1 is up-regulated upon activation of T cells and B cells (12). The ligand PD-L1 is broadly expressed by immune cells including T cells, B cells, dendritic cells and macrophages, and in non-lymphoid tissues including on tumor cells or stromal elements in the tumor microenvironment (TME) (11, 13). Recent evidence suggests that CD28 is also a primary target for PD-1 (14, 15).

Intracellularly, both PD-1 and CTLA-4 signal *via* SHP2 and converge to inhibit downstream P13K signaling (16, 17). Although it is reported that B cells express PD-1 and PD-L1, these cells have not been recognized as a primary target for anti-PD-1 therapy. CTLA-4 also acts extracellularly to clear the CD28 ligands CD80/86 from surrounding cells, including APCs, by trans-endocytosis *in vivo*, further inhibiting T-cell activation (18). While B cells are also known to express CTLA-4 ligands CD80/CD86, again they are not considered the key target cells of anti-CTLA-4 therapy (10).

## Overview of B Cells in Melanoma, in the Tumor Microenvironment, and Tertiary Lymphoid Structures

Besides constituting an amalgamation of malignant cells, the tumor microenvironment (TME) can be thought of as a distinct complex “organ” which contains an array of different cells, such as immune cells, tumor-associated vasculature and lymphatics, fibroblasts and pericytes (15). The immune cell infiltrates are of special importance as they often correlate with anti-tumor activity and have been reported to be associated with a more favourable prognosis in some settings. Melanoma is a classically highly immunogenic tumor (19) and lymphocyte infiltrates in general have been shown to have positive prognostic value in patients with primary cutaneous melanoma (20). Immune cell subsets which are correlated with increased patient survival outcome include CD8+ memory T cells, CD4+ T-helper cells (21), B cells (22) and NK cells (23). Immune cell infiltrates harbor the potential to exert cytotoxic anti-tumor activity, which may be exacerbated by checkpoint inhibitor immunotherapy.

Lymphocyte activation typically occurs in secondary lymphoid structures which include lymph nodes, the spleen and mucosal-associated lymphoid tissue (24). During chronic inflammation, TLS may transiently form, which confer sites enriched in B and T lymphocytes and dendritic cells present from disordered compositions to more structured and often highly specialized germinal center-like formations (25). As tumors share many of the characteristics of chronic inflammation, it is unsurprising that TLSs have been identified in a range of cancers adjacent to the tumor site, including in 26% of metastatic melanomas (26). Importantly, the presence of TLS has been shown to be associated with a more favorable prognosis (27, 28) and the promotion of immunotherapy response (discussed below) (29). There is compelling evidence of ongoing B cell maturation in these sites, driving clonal amplification, class switching and somatic hypermutation (SMH). It is therefore clear that intralesional B lymphocyte structural organization and the interaction with other lymphocytic cell subsets within TLS could be of particular importance to overall B cell-mediated anti-tumor activity.

The majority of melanoma lesions contain significant populations of B cells, usually localized within the tumor stroma (one study has reported that 86% of primary melanomas harbor at least ten CD20^+^ stromal B cells per mm^2^ tumor (30). Furthermore, tumor infiltrating B lymphocytes TIL-B in primary lesions make up an average of 10% of the total infiltrating lymphocytes (31–33) and can be found adjacent to various other immune cell subsets. CD22^+^ B cells are enriched in melanoma lesions compared to healthy skin, and CD20 and CD22 mRNA expression is further enhanced with metastasis (34, 35), indicating the presence of a pronounced humoral immune response in melanoma.

These TIL-Bs have been shown to contribute to anti-tumor immunity *via* antibody responses to melanoma-associated antigens. In one study, 28% of melanoma patient-derived B cell cultures were capable of binding to a wide range of antigens expressed by melanoma cell lines, as opposed to just 2% of those derived from healthy individuals, and this was shown to further increase with metastatic disease (36). In concordance, plasma cells in melanoma lesions were shown to be polyclonal and to predominantly express IgG and IgA (37) implicating antigen reactivity and class switching, and thus a possible melanoma-reactive B cell immune response. Secreted immunoglobulins, especially of the IgG1 subclass, may be able to exert potent tumoricidal effects *via* ADCC, as demonstrated *in vitro* using a patient-derived monoclonal antibody (36). ADCC however is just one of several effector mechanisms mediated by secreted immunoglobulin. A neutralizing anti-human growth factor (HGF) antibody has been shown to suppress tumor growth *in vitro* (38). B cells may also act as antigen presenting cells, contributing to T-helper cell activation and anti-tumor effector mechanisms (39). Finally, activatory B cells may facilitate anti-tumor immunity *via* the release of pro-inflammatory cytokines such as IL-2, TNFα and IFN*γ* (40, 41). TNFα produced by B cells may control the development of follicular dendritic cells, the formation of B cell follicles and T cell-dependent antibody responses (42). Collectively, several studies therefore support the presence of an antigen-specific B cell immune response to melanoma.

In accordance with expectations arising from these anti-tumor properties, the percentage of tumor-infiltrating and peritumoral B cells have been found to positively correlate with more favorable patient survival in the vast majority of melanoma cohort studies (22, 43, 44). However, some studies have in contrast found no significant association between B cell infiltration and overall patient survival (31), and even a correlation of B cell infiltrates with a poorer prognosis (33). These discrepancies may arise from differences in experimental design and analyses (*e.g.* using raw B cell counts *versus* percentage of infiltrating populations) or may reflect the confounding presence of immunosuppressive regulatory B cells.

## B Cell Subsets In Melanoma and the Role of Bregs

Several studies have demonstrated the enriched presence of various differentiated B cell phenotypes such as memory, plasmablasts (PBs) and plasma cells within melanoma tumors. Elevated percentages of PBs (short-lived cells and part of the rapid antigen response) have been reported in the circulation of patients with melanoma compared to blood from healthy individuals. PBs are short-lived cells and part of a rapid antigen response. Although PBs have a potentially positive role in melanoma, the functional plasticity of these cells may result in transient switching to a “regulatory” pro-tumor phenotype owing to expression of TGFβ and IL-10 by this cell subset (41).

IL-10-producing “regulatory” B (B10) cells (45) have been shown to be functional in murine models of tolerance (46), in preventing chronic inflammation (47–50) and in curbing anti-tumor immune responses (51). In mouse models, Bregs appear to be derived from either BII (follicular) or Marginal Zone (MZ) cells. A CD1d^+^ CD19^++^ regulatory B cell subset has been identified in mice which strongly resembles MZ cells and protects against colitis, through interactions with Tregs (52). These observations not only demonstrate that these cells play a crucial pathogenic role in health and disease, but also that B cells can contribute to immune outcomes in general *via* their polarized cytokine expression.

Studies in mouse models of skin cancer suggest that there are certain circumstances where B cells can exert a distinctly immunosuppressive pro-tumor effect (53). A subset of “tumor-evoked” Bregs has been shown to facilitate the metastasis of breast cancer tumors through the induction of Tregs *via* TGF*β* secretion (54). Importantly, tumor-associated antigens such as 5-lipoxygenase (breast cancer (55); and placental growth factor (glioblastoma (56); have been shown to induce IL-10-production in tumor-infiltrating B cells in murine models. It may therefore be possible that B cells in the tumor-microenvironment are coerced into developing a regulatory phenotype (TGFβ^+^ and IL-10^+^), and that tumor-derived antigens in general could induce B cell IL-10-expression (57). As expected, Bregs have been shown to directly contribute to tumor-progression as a result of their anti-inflammatory properties (58).

At present there is a lack of substantial information relating to the role of immunosuppressive IL-10-producing B cells in affecting tumor progression in humans. Perhaps the most significant study showed that Granzyme-B-expressing IL-10+ B cells induced by T cells IL-21 secretion infiltrate solid tumors of breast, cervical, and ovarian cancer patients and inhibit T cell responses (59). In addition, IL-10+ transitional B cells have been shown to be up-regulated in gastric cancer patient peripheral blood and tumors and are functionally capable of suppressing Th1 cytokine secretion by T cells and inducing Tregs (60).

The current evidence for regulatory B cells in melanoma tissue microenvironments rests upon a study demonstrating that adoptive transfer of B1a B cells (possessing an IL-10+ regulatory phenotype) into wild-type mice significantly exacerbated B16F10 melanoma growth, illustrating that IL-10-producing B cells can directly contribute to melanoma tumor-progression (61). A separate murine model of squamous cell carcinoma obtained similar conclusions (62). Finally, Bregs have been shown to supress cutaneous inflammation in a mouse model of psoriasis-like inflammation (63). To date, there have been no investigations into the role of IL-10-producing B cells in patients with melanoma.

Tumor-infiltrating regulatory B cells therefore represent a potential mechanism of tumor-mediated immune escape that in certain conditions may outweigh antigen-presentation and anti-tumor antibody-mediated effector mechanisms to skew the overall impact of B cell infiltrates towards a neutral or even a negative effect, and mandates studies into the induction of regulatory B cells in melanoma patients and correlation with overall survival.

## Influence of the Th2-Biased Melanoma Microenvironment on Pro-Tumor Properties of B Cells

Consistent with the contributions of regulatory B cells, the microenvironments of melanoma solid tumors are characteristically considered as harboring Th2-biased cytokine expression profiles (64), typical of chronic inflammatory conditions. These microenvironments typically confer pro-tumor properties including promoting angiogenesis and inhibiting cell-mediated responses, such as those mediated by cytotoxic T-lymphocytes (CTLs). The classic Th2 cytokine is interleukin-4 which, in addition to interleukin-13, may induce tumor clearance (65). However, IL-10 produced by Bregs, Tregs and M2-type macrophages in the tumor microenvironment can, in addition to its potent suppressive effects upon CTLs, trigger a modified or alternative Th2 response by inducing B cell IgG4 subclass switching in the presence of IL-4 (66).

Indeed, it has been shown *in vitro* that these Th2-biased microenvironments favor alternatively activated humoral immunity which confers a shift in B cell antibody expression towards IgG4 (67). Early evidence has accordingly demonstrated that IgG4 serum levels are dysregulated in melanoma patients, particularly in advanced disease settings (68). IgG4 subclass antibodies are structurally distinct to IgG1 antibodies and confer a weaker ability to bind and stimulate effector cells such as macrophages to initiate ADCP and ADCC through their Fc-gamma receptors (69). It has been shown that these antibodies compete with IgG1 for Fc-gamma receptor sites resulting in a reduced degree of immune cell activation and tumor-cell killing when secreted in large quantities (34, 67). Recent evidence also suggests that Fc–Fc interactions between IgG4 and cancer-specific IgG1 antibodies may confer an important aspect of tumor immune evasion (70). Interestingly, the same study also demonstrated that the IgG4 subclass CPI antibody Nivolumab participated in Fc-interactions with IgG1 subclasses and promoted cancer growth in a murine model of colon cancer. The pro-tumor role of IgG4 subclasses may appropriately explain observations of possible hyperprogressive disease seen in some CPI patients.

IgG4 is therefore a negative prognostic indicator of patient overall survival, and these Th2 biased microenvironments offer a form of immune evasion which is mediated by IL-10 (71, 72). Interestingly, recent evidence has also revealed that an IgG4-expressing CD49b+ CD73+ B cell subset expressing proangiogenic cytokines including VEGF is up-regulated in the circulation of melanoma patients, highlighting an additional and previously unknown mechanism by which tumor-infiltrating B cells may contribute to tumor progression (73).

## The Role of B cells in Checkpoint Inhibitor Therapy Responses

**Figure 2:**
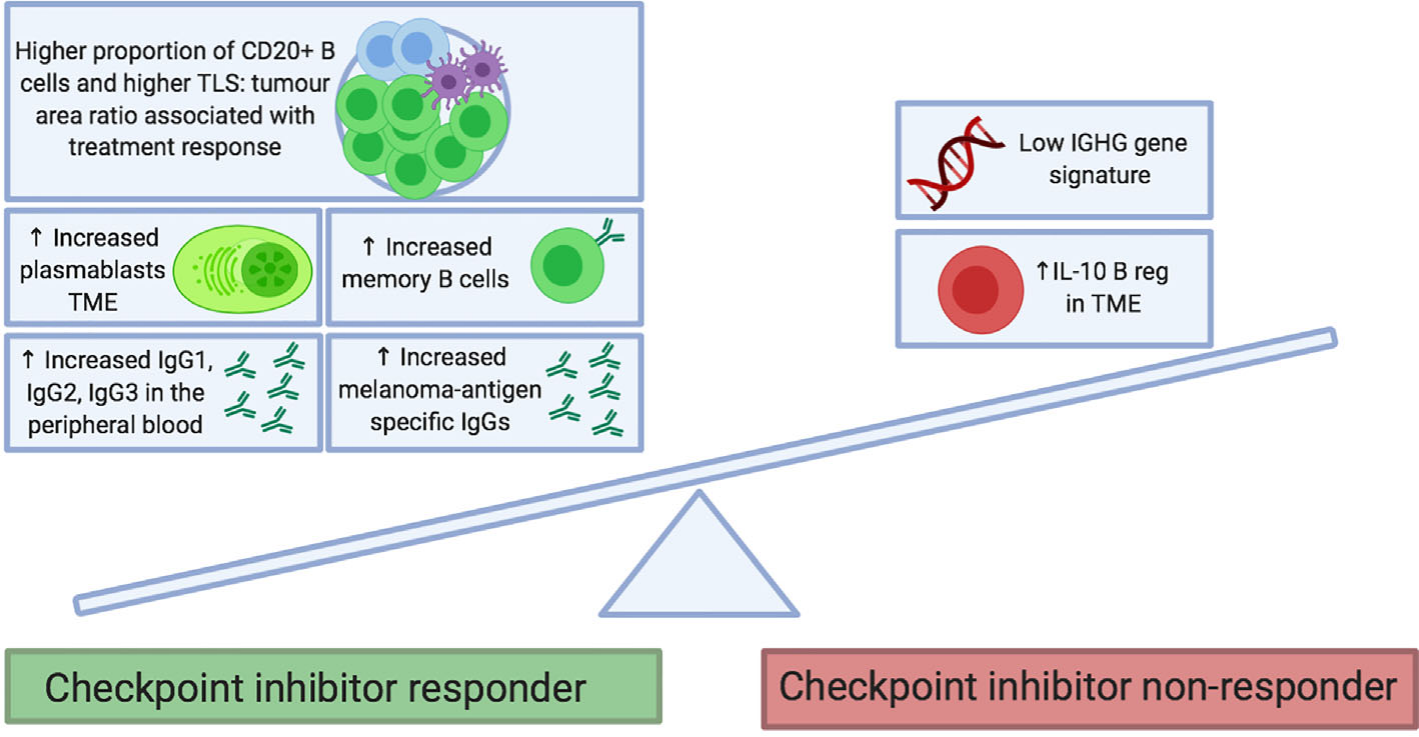
Proposed B cell features at baseline that predict response to checkpoint inhibitor treatment. B cell phenotypes enriched in tumors and the circulation of patients before treatment may be predictive of clinical response to checkpoint inhibitor therapy. Features include the presence of memory B cells (*e.g.* Ipilimumab monotherapy and combination of Ipilimumab and Nivolumab) and plasmablasts (*e.g.* Ipilimumab and Nivolumab monotherapies). In the peripheral blood, immunoglobulin isotypes IgG1, IgG2 and IgG3 have been associated with response to Ipilimumab and Nivolumab monotherapies. Melanoma-antigen specific IgG antibodies have been found to be increased in responders to therapy (monotherapy with Ipilimumab, Pembrolizumab and Nivolumab; as well as with combination of Ipilimumab and Nivolumab). Conversely, enrichment of tumors with IL10+ regulatory B cells and an absence of an IGHG gene signature have been associated with a poor response to Ipilimumab. Fc*γ*R, Fc*γ* receptor; NK, natural killer; TCR, T cell receptor.

Checkpoint inhibitors are designed to regulate T cell effector functions; however, there is growing interest in the contribution of B cell responses to patient outcomes. This has been examined in several recent studies; the findings are summarized in **Table 1** and **Figure 2**.

**Table 1:** Summary of current evidence of a humoral immune response in patients treated with checkpoint inhibitor therapy.

Sample type	Time point	CPI used	Target	B cell and antibody responses	Reference
Tumor	Baseline	Ipilimumab	CTLA-4	▪ IL10+ Breg enrichment found in non-responders to anti-CTLA-4 therapy	(22)
				▪ Absence of IGHG gene signature enriched in non-responders to anti-CTLA-4	
		Nivolumab	PD-1	▪ IL10+ Breg presence did not correlate with treatment response to anti-PD-1	
				▪ IGHG gene signature did not correlate with treatment response to anti-PD-1	
Tumor	Baseline	Ipilimumab & Nivolumab combination	CTLA-4 & PD-1	▪ For all treatments increased B cell frequency found in the tumours of responders to both combination therapy and anti-PD-1 monotherapy in both baseline and early on treatment samples	(29)
		Nivolumab	PD-1		
	Early on treatment	Ipilimumab & Nivolumab combination	CTLA-4 & PD-1	▪ For all treatments higher density of CD20+ B cells and TLS and a higher TLS:tumour area ratio in early on-treatment samples from responders versus non-responder	
		Nivolumab	PD-1	▪ For all treatments increased frequency of switched memory B cells found in responders at baseline and early on treatment	
Tumor	Baseline	Ipilimumab	CTLA-4	▪ B cells found to localise within TLS	(27)
				▪ TLS gene signature present in responders’’	
				▪ TLS structures associated with CD8+ T cells	
Peripheral blood	After 1 year of treatment	Ipilimumab	CTLA-4	▪ High plasmablast numbers seen in patients responding to anti-CTLA-4	(74)
				▪ Sequenced plasmablast antibodies in patients who had not progressed after 1 year had somatic hypermutation, class switching and clonal expansion showing an active humoral response	
Tumor	Baseline	Nivolumab	PD-1	▪ Plasmablast-like phenotype (CD19+CD20+CD38+CD138-) found to be most common B cell phenotype in TME	(41)
				▪ Tumour induced plasmablast population (TIBP) defined using melanoma secretome	
				▪ Increased expression of TIPB gene signature in baseline samples associated with improved response to Nivolumab	
Peripheral blood	Baseline	Nivolumab	PD-1	▪ Melanoma-antigen specific antibodies to: MDA (TRP1/TYRP1, TRP2/TYRP2, gp100, MelanA/MART1) and the Cancer-Testis antigen NY-ESO-1 higher in responders to all treatments	(75)
		Pembrolizumab	PD-1		
		Ipilimumab	CTLA-4		
		Ipilimumab & Nivolumab combination	CTLA-4 & PD-1	▪ Melanoma-antigen specific IgGs higher in responders at baseline compared with non-responders for all treatments ▪ Melanoma-antigen specific IgGs in patients were mainly IgG1 and IgG2	
Peripheral blood	Baseline	Nivolumab	PD-1	▪ For all treatments high titres of IgG, IgG1, IgG2 and IgG3 showed a positive correlation with progression-free survival (PFS)	(76)
		Pembrolizumab	PD-1		
		Ipilimumab	CTLA-4		
		Ipilimumab & Nivolumab combination	CTLA-4 & PD-1	▪ For all treatments high titre of IgG2 showed a positive correlation with overall survival (OS)	
Peripheral blood	Baseline	Ipilimumab & Nivolumab combination therapy	CTLA-4 & PD-1	▪ Overall decline in B cell numbers but increased plasmablast and CD21^lo^ B cell subsets corresponded to development of high grade immune related adverse events (irAE)	(77)
	Early on-treatment				
				▪ Changes in other immune cells not significantly associated with frequency or severity of irAE	

### Tumor-Infiltrating B Cell Signatures in Relation to CPI Treatment Response

Further evidence supports the notion that specific B cell subsets may hold prognostic value in CPI treatment response in advanced melanoma. B cell receptor repertoire and B cell phenotype were analyzed in 473 cutaneous melanoma specimens. The absence of B cell immunoglobulin heavy chain *γ* (IGHG) gene was found to correlate with lack of response to anti-CTLA-4 but this was not shown in the context of anti-PD-1 therapy (22). In addition, a higher proportion of IL-10 secreting B cells was associated with lack of response to anti-CTLA-4, but was not correlated with response to anti-PD-1 therapy (22). A phase 2 trial of neoadjuvant CPI [neoadjuvant Nivolumab (n = 11) *versus* neoadjuvant Nivolumab plus Ipilimumab (n = 12)] (78) also highlighted the importance of B cells in CPI response. While baseline tumor-infiltrating B cell counts did not differ significantly between responders and non-responders in this small sample size, early on-treatment tumor samples showed increased tumor-infiltrating B cell frequency (as defined by CD20 immunostaining) in treatment responders compared with non-responders. Helmink et al. used the same cohort and performed bulk RNA sequencing from baseline and early on-treatment tumor biopsy samples (29) and found that B cell markers were the most differentially expressed genes in responders to therapy compared with non-responders. They identified significantly increased clonal counts for both immunoglobulin heavy and light chains and increased BCR diversity in responders than in non-responders, which suggests an active role for B cells in anti-tumor immunity. Pathways up-regulated in responders as compared to non-responders included those consistent with increased immune activity such as CXCR4 signaling, cytokine receptor interaction and chemokine signaling pathways. Phenotyping of B cells in tumors identified significantly higher frequency of memory B cells in responders *versus* non-responders at baseline and early on-treatment. They corroborated their findings in a further cohort of 18 patients with melanoma treated with adjuvant or neoadjuvant CPI. Unlike tumor-derived B cells, a peripheral blood B cell phenotype was not found to correlate with treatment outcome (29). Since most samples in this study were blood or metastatic lymph node deposits, the cohort was relatively small and the samples were sourced either at baseline or early on-treatment; these findings would benefit from evaluations of larger and more comprehensive studies.

Furthermore, in a cohort of 39 patients with advanced melanoma, early changes were observed in peripheral B cell subsets upon one cycle of combination CPI therapy (Ipilimumab and Nivolumab): particularly a significant decrease in circulating B cells in patients who underwent combination CPI as opposed to those treated with a monotherapy anti-CLTA-4 or anti-PD-1 (77). No significant differences were found between naïve and memory B cell sub-populations. Despite the overall decline in B cell numbers, a marked increase was observed in the density of: class-switched memory B cells, plasmablasts and CD21^lo^ B cell sub-populations (77). Of note, significant changes in the latter were only exhibited in patients who underwent combination or anti-CTLA-4 treatment. This is consistent with findings from a previous study whereby loss of circulating B cells and increase in CD21^lo^ B cells were reported in patients treated with an anti-CTLA-4 agent (79). Detailed evaluation of the CD21^lo^ B cell compartment revealed equal concentrations of naïve and memory B cells at baseline, but moderate increase in memory B cells after combination checkpoint inhibition. Furthermore, B cell receptor sequencing demonstrated that CD21^lo^ B cells possessed higher: clonality, maximal clone frequency, and SHM frequency. Therefore, CD21^lo^ B cells were shown as a phenotypically distinct B cell subset present in melanoma patients, which increased in frequency following combination checkpoint inhibition and suggest an active B cell response in the TME. PD-1 expression on the CD21^lo^ B cells was higher than on other B cell subsets, suggesting that this population may be specifically modulated by anti-PD-1 therapy. This study failed to find an association between B cell changes and clinical response. The extrapolation of these findings may be limited due to the small sample size and the fact that on-treatment peripheral blood samples were only taken at an early time point after commencement of CPI therefore may not be representative of the full response.

### CPI-Associated Modulation of Plasmablasts

There is some evidence that plasmablasts (PB) may be directly modulated by CPI. Circulating plasmablast levels were significantly enhanced in patients undergoing treatment with anti-CTLA-4 immunotherapy (74). When immunoglobulins produced by the PBs were sequenced, there was evidence of clonal expansion, SMH and class switching, indicative of an active B cell response (74). Immunostaining of 41 metastatic melanoma specimens revealed that a plasmablast-like phenotype (CD19+CD20+CD38+CD138−) is the most frequently observed B cell subset in the TME (41). Furthermore, circulating B cells could be induced to differentiate into plasmablast-like cells when exposed to autologous melanoma secretomes *in vitro* (41). In this study tumor-associated B-cells also expressed T-cell and macrophage chemoattractant mediators (CCL3, CCL3L1, CCL4, CCL5, CCL28, and CXCL16) suggesting that B-cells can orchestrate the immune response in the TME by modulating other cell populations. Increased expression of immunosuppressive cytokines IL-10 and TGFβ1 was reported in plasmablast-like and memory B cell sub-populations, consistent with a potential regulatory role. However, these signatures may also be associated with treatment response: proteomics and RNA-seq data were used to define a tumor-induced plasmablast-like population (TIBP) functional signature, and this signature was evaluated in a historical dataset from patients treated with Nivolumab. In this analysis, the presence of this TIBP signature at baseline correlated with better response to anti-PD-1 treatment (41).

### A Role for Tertiary Lymphoid Structures

The importance of TLS in response to CPI has also been investigated. Helmink et al. delved deeper into the localization of B cells within their original cohort of 23 tumors and identified a higher density of CD20+ B cells in TLS and a higher TLS:tumor area ratio in early on-treatment samples from responders *versus* biopsies from non-responders (29). They also illustrated that CD20 B cells often co-localized with CD4, CD8 and FOXP3 T cells. A second study also identified CD20 B cells in 25% of 177 melanoma specimens (27). CD20 B cell clusters were surrounded by CD4 T cells. Increased expression of known molecular markers of TLS (CXCL13, CXCR5 and DC-LAMP) was described and immunostaining confirmed the co-localization of CXCL13 and CXCR5 with the CD20 B cell marker. A TLS gene signature in melanoma was defined and integrated into four historical melanoma cohorts treated with CPI. This TLS gene signature at baseline was associated with significantly increased survival with anti-CTLA-4 therapy, and this was independent of the tumor mutational burden which may be a predictor for TLS formation (27). The role of B cells in TLS in response to CPI has also been highlighted in a third study in the treatment of sarcoma (80). In this, 213 soft tissue sarcomas were stratified according to the composition of the TME. Those with highest density of B cells and TLSs were associated with the highest response rate to CPI. The study identified several immune cell-related gene signatures and the only one significantly associated with extended overall survival was the B cell signature (80).

Taken together these studies highlight that B cell phenotype, localization with TLS and B cell gene expression may be directly modulated by CPI and could be relevant to treatment outcomes. The differential results seen with different agents and combination therapy likely reflect the distinct mechanisms of action of each treatment (81).

## Antibody Responses in Melanoma

A key feature of B cells is their ability to produce antibodies. B cells in the tumor can undergo class switching and affinity maturation, producing tumor-specific or non-specific antibodies of different isotypes (24) and could exert anti-tumor or pro-tumor roles (82). Human antibodies are tetrameric glycoproteins formed by two heavy and two light polypeptide chains. Heavy and light chains assemble in a Y shaped structure, defined by a Fab region (formed by two identical Fab fragments, composed by the light chain and part of the heavy chain), an Fc region (formed by the constant portion of the two heavy chains) and a hinge region (joining Fab and Fc regions). The variable regions of heavy and light chains are assembled to form two identical antigen binding sites. The heavy chain constant region determines the isotype of the antibody, with a characteristic hinge and Fc region. The hinge region is responsible for the flexibility of the Fab arms which can affect both antigen binding and effector function and for some isotypes, such as IgG4, these can be involved in a process called Fab Arm Exchange, which results in bispecific (IgG4) antibodies (72). The Fc region is involved in the binding to Fc receptors (FcRs) and C1q (complement) and is responsible for antibody mediated effector functions such as ADCC, ADCP and CDC (81).

Depending on their isotype and specificity, antibodies can have opposite roles in the tumor-associated immune response. Different antibody isotypes have different affinity for FcRs and C1q and consequently have different abilities to trigger effector functions. Tumor-specific antibodies with high affinity for FcRs and C1q (such as IgG1) have the potential to engender anti-tumor effects, mediating ADCC, ADCP, CDC or to support antigen presentation by mediating the uptake of tumor antigens by APCs such as macrophages and dendritic cells (82). Furthermore, membrane bound tumor-specific antibodies on the surface of B cells can engage with tumor cells and trigger B cell mediated cytotoxicity. Evidence of B cell mediated cytotoxicity *via* granzyme B and TRAIL has been shown in hepatocellular carcinoma (83). IgG class antibodies have been found to be tumor-specific, and this has been reported in melanoma (75) and other cancer types and has been correlated with a favorable prognosis (84–87).

Tumor-associated antibodies can also have either pro-tumor effects or their anti-tumor effects may be regulated by tumors. Tumor-specific antibody-mediated immune cell activation and anti-tumor cytotoxicity may also be impaired by expression of inhibitory Fc receptors (Fc*γ*RIIbs) in the TME and downstream immunoreceptor tyrosine-based inhibitory motif (ITIM) domain signalling (88, 89). Tumor-specific antibodies may be expressed as isotypes (such as IgG2 or IgG4) with poor affinity for activating FcRs or complement. These isotypes can be ineffective in triggering anti-tumor responses like ADCC, ADCP, CDC and antigen presentation (72). Furthermore, IgG4 antibodies can engage the inhibitory receptor Fc*γ*RIIb. This might further impair anti-tumor responses, blocking IgG1 mediated effector function (67). Tumor-associated antibodies not specific for the tumor can also serve as a decoy blocking other potentially immuno-activating and tumor-reactive antibodies from engaging with immune effector cells. This can contribute to pro-tumor effects such as ineffective T cell mediated responses or B effector functions. It is also possible that tumor-specific antibodies accumulating at the tumor site over time forming immune-complexes, promoting chronic inflammation and immunosuppressive myeloid cell phenotypes (82).

Isotypes like IgG1 and IgG3 have high capacity to trigger ADCC and facilitate complement activation. The IgG1 isotype is usually associated with anti-tumor responses while TCGA RNA-seq data analysis of melanoma samples showed that IgG3 is either neutral or is associated with a negative prognosis. This could be explained by the fact that IgG3 has a shorter half-life (1 week) compared to other isotypes (IgG1, 2 weeks). Furthermore B cells expressing IgG3 isotype antibodies usually undergo less antibody SHM, and therefore IgG3 antibodies may have lower affinity for the target antigen while still able to occupy FcRs (90). IgG2 has poor ability to bind FcRs and C1q. Evidence of antibody responses switched to IgG2 has been found in melanoma (26). IgG4 has poor ability to bind activating FcRs while able to bind the inhibitory receptor Fc*γ*RIIb and has complement-activating ability. Evidence of IgG4 antibody responses has been found in the melanoma TME, and serum IgG4 has been associated with poor prognosis in melanoma (36, 67, 71).

TCGA RNA-seq data analyses also suggest that IgD, IgE, and IgA antibodies may be associated with poor prognosis in melanoma (90). One possible explanation is that IgD can bind basophils resulting in the production of cytokines like IL-4, IL-5, IL-13, BAFF, and APRIL which may support class switching to IgA and IgE and a Th2 immune response. Furthermore, the presence of IgE antibodies is usually associated with IL-4, which can further support a Th2 immune response. IgA antibodies have been found in melanoma and are associated with poor prognosis. This could be explained by the immune-suppressive phenotype of IgA+ B cells, expressing IL-10 and PD-L1 and by the ability of IgA+ B cells to support the expansion of T regulatory (Treg) cells which in turn secrete TGF*β* supporting IgA class switch. Melanoma-associated IgA has been found to correlate with poor clonality, suggesting that, in the tumor, class switching to IgA is a consequence of inflammation and not of an antigen-driven response, and that tumor-associated IgA could be non-tumor specific (82).

## Associations of B Cell Expressed Antibodies With CPI Treatment

Based on mounting evidence in support of potential roles of various antibody isotypes in melanoma, it is not surprising that antibody responses have been studied and are thought to correlate with outcome of CPI therapy. In the context of B cells in HIV infection, blocking of PD-1 has been shown to increase viral antigen-specific antibody responses (91). This may lend merit to the notion that PD-1-specific CPI could improve tumor-specific antibody responses (92).

One study investigated whether IgG antibody and isotype levels correlated with anti-tumor response and survival following CPI therapy. IgG subclass analysis was performed on serum samples at baseline from 49 patients with melanoma treated with anti-PD1 antibody (Nivolumab or Pembrolizumab (86%), Nivolumab plus Ipilimumab (10%), or Ipilimumab (4%). A positive correlation with progression-free survival (PFS) was found for high titers of total IgG, IgG1, IgG2, and IgG3, while a positive correlation with overall survival (OS) was found to be significant only for the IgG2 subclass (76). This result is quite interesting since IgG2 is usually not associated with anti-tumor immune responses due to its poor ability to trigger ADCC, ADCP, CDC and facilitate antigen presentation, but may reflect activation of a tumor antigen-specific response triggered by CPI.

A different preliminary study compared the antibody sequence repertoires of 26 patients with melanoma treated with Pembrolizumab (9), Ipilimumab (8) or Nivolumab, and Ipilimumab combination (9). It was found that long-term responders had higher levels of SHM after initiation of treatment, and on-treatment compared to the non-responders. Comparison of lineages between patients identified antibodies with high sequence similarity suggesting these antibodies may have arisen from convergent selection, *i.e.*, different patients raising antibodies against shared or similar epitopes. Moreover, when antibody lineages were analyzed and compared among patients, antibodies with high sequence similarity were found, suggesting these antibodies may have arisen from convergent selection and that the patients may be producing antibodies against shared epitopes. Of note, IgG2 was the most frequent isotype of these antibodies. Furthermore IgG2 was higher in responders compared to non-responders (93).

In a different study, melanoma patients treated with CPI (monotherapy with Nivolumab, Pembrolizumab or Ipilimumab, or the combination of Nivolumab and Ipilimumab), the responder group had higher titers of antibodies specific for the tumor-associated antigens MDA (TRP1/TYRP1, TRP2/TYRP2, gp100, MelanA/MART1) and the Cancer-Testis antigen NY-ESO-1 at baseline, compared to non-responders. Serum analyses showed that NY-ESO-1, TRP1/TYRP1, and TRP2/TYRP2 specific antibodies consisted of several IgG subclasses while the IgG antibodies for MelanA were mostly IgG1 and the antibodies for gp100 were of the IgG2 isotype (75). Interestingly, none of these antibodies were IgG4, supporting the fact that IgG4 antibodies are not associated with a good prognosis in melanoma patients (67, 71, 75, 76).

In patients with metastatic melanoma who were treated with anti-CTLA-4 combined with anti-angiogenic VEGF-A-targeted treatment, there was an increase of antibody titers recognizing the immunoregulatory protein Galectin-1 (Gal-1). Anti-Gal-1 antibody titers in turn correlated with better disease outcome. Higher frequencies of complete or partial responses and improved overall survival were seen in these individuals. Gal-1 has been reported to be up-regulated in many tumor types including melanoma and is usually associated with poorer survival, contributing to tumor growth, angiogenesis, metastasis, and immune evasion (94).

Taken together, these results suggest that checkpoint blockade-associated restoration of T cell activity may also contribute to increasing the effectiveness of the humoral response and highlights the importance of the expressed antibodies as part of the anti-tumor activity. Furthermore, these studies suggest that melanoma-specific antibodies in pre-treatment sera may be promising indicators of CPI immunotherapy response and require further study.

## Correlations With Immune-Related Adverse Events in CPI Therapy

Activation of immune responses as a result of checkpoint inhibition can lead to compromised immune self-tolerance and can consequently result in the development of irAEs, toxicities that mimic autoimmunity. The nature and frequency of irAEs vary among CPI agents and can include effects on the gastrointestinal tract (colitis), liver (hepatitis), lung (pneumonitis), and endocrine systems, including hypophysitis and insulin-dependent diabetes. The effects of anti-CTLA4 associated irAEs are generally more severe than those from anti PD-1 inhibition. Whether irAEs represent *de novo* events or whether they represent an unmasking of underlying immune mediated disease remains unclear (6).

Modulation of B cell phenotype has been correlated with irAE in patients undergoing CPI therapy. A recent study (described above) demonstrated an overall decline in B cell numbers but increased plasmablasts and CD21^lo^ B cell subsets in patients given combination checkpoint inhibition (**Table 1**) (77). These CPI-induced B cell changes positively correlated with frequency and severity of irAEs. Furthermore, patients with altered B cell populations were more likely to develop multi-organ immunotoxicity than those without, with these changes preceding toxicity by a median of 3 weeks.

B cells are proposed to contribute to autoimmunity *via* multiple mechanisms including as antibody secreting cells, cytokine producers, APCs, and immunoregulators. Given the important role of the checkpoint pathways in regulating immune homeostasis, it is not surprising that activation of the CTLA-4 pathway has been extensively studied in the treatment of autoimmunity. Specifically Abatecept, a fusion protein of CTLA-4 and IgG1 Fc portion (CTLA-4-Ig), is approved for abrogating immune overactivity in rheumatoid arthritis (95–97). CTLA-4-Ig is able to directly bind CD80/86; therefore, preventing CD28 binding and T cell co-stimulation. B cells are known to express CD80/86 and have been shown in mouse models and *in vitro* to be a direct target for CTLA-Ig. In synovial biopsy samples, CTLA-4-Ig has been shown to decrease B cell infiltrates (95). Humoral responses to T cell dependent and T cell independent antigenic stimulation are also suppressed by CTLA-4-Ig *in vivo* (98, 99). These insights from autoimmunity may also have relevance to our understanding of how CPI modification of B cells may induce irAE.

The presence of autoantibodies in CPI-induced irAE has been detected in a similar fashion to those seen in autoimmune disease in the absence of CPI treatment. For example some patients suffering with CPI-induced hypothyroidism have raised titers of anti-thyroperoxidase antibody and/or anti-thyrotropin receptor antibodies (100, 101). However, as these autoantibodies are not universally detected in CPI thyroid disorders, it is unclear whether they play a causative role. Similar observations have been made in a range of CPI-induced autoimmune phenomena including cases of myasthenia gravis (102), type 1 diabetes mellitus (103) and autoimmune hemolytic anemia (104). One study analyzed 23 common autoantibodies in baseline peripheral blood and post CPI treatment in 133 melanoma patients treated with Ipilimumab (105). This showed that 19% of patients developed autoantibodies on-treatment and a trend of association with onset of irAE which did not reach statistical significance. In this study, organ-specific autoantibodies did not correlate with irAE.

CPI-induced irAEs present a clinical challenge for patient selection and can lead to treatment discontinuation. Insights into the complex balance between activation and suppression of B cell subsets may infer consequences for both treatment response and irAE. In addition, the potential contributions of autoantibodies to irAE warrant further investigation and may be of direct relevance to B cell biology in the context of CPI.

## Harnessing The Humoral Immune Response in Melanoma Immunotherapy

Data from several studies suggest that B cells can play multi-faceted roles in the immune response to melanoma and that melanoma-associated immune evasion mechanisms involve modulating B cell immunity in addition to suppressing T cell activation. Despite the impressive efficacy demonstrated by CPI, a large proportion of patients fail to respond and/or develop irAEs (4, 106, 107). Modulated B cell and antibody repertoires could either obstruct or facilitate the efficacy of CPI, depending on an array of factors in the TME. Further research identifying specific cellular surface markers and methods to clearly dissect the various B cell phenotypes will help improve our understanding of B cell immunity in melanoma and whether and how this is altered by CPI. This may then be exploited in clinical trials to help identify therapeutic strategies which can be combined with CPI to improve response. This may include the targeting of B cells; a pilot study using Rituximab (anti-CD20) to deplete B cells in melanoma patients increased the median time without recurrence from 6 to 42 months (108). On the other hand, B cell depletion therapy may have contrasting effects dependent on the stage of melanoma in mouse models (41). Given the newfound appreciation of the complexity of the TIL-B sub-populations, a more effective approach may involve depleting specific pro-tumor subsets, such as Bregs and IL-10+ B cells, while promoting anti-tumor subsets which could be combined with CPI as an adjuvant (24). PD1-PD-L1 engagement plays a role in suppressing B cell-mediated T cell activation, influencing CPI efficacy. Inhibition of PD-1 expressed on B cells may also be a mechanism through which CPI directly modulates B cell responses.

The targeting of the immunosuppressive TME cytokines IL-10, VEGF or TGF-*ß* may effectively reinstate robust anti-tumor immunity by preventing IgG1 to IgG4 class switching and promoting anti-tumor B cell subsets to engage in tumor rejection. The hostile TME may be taken into consideration in the engineering of therapeutic antibody candidates, with the aim of potentially producing antibodies more resilient to immune suppression. CPI antibodies could additionally be engineered to have reduced binding to inhibitory Fc receptors, increased binding to activatory Fc receptors, or consider alternative isotypes such as IgE which has been implicated to improve immune surveillance in melanoma (109–111).

Aside from clinical response, irAEs are a major challenge of CPI in melanoma, as 96% of patients receiving combined CPI experienced at least one irAE in the CheckMate067 trial (4). Immune monitoring for early alterations in B cells following CPI may hold some promise in determining those at risk of developing irAEs. If confirmed to contribute to irAEs, therapies targeting plasmablasts and CD21^lo^ B cells could be explored to reduce the risk. Serum and melanoma IgG4 and IgG4+ B cell levels may also be monitored, and therapies targeting these subsets could be conducted prior to CPI to establish a ‘hot’ tumor more likely to respond to CPI. Throughout CPI, B cell and antibody levels may be monitored to determine modulation and predict prognostic outcomes, and spikes may be countered through further depletion methods. Similar strategies could be conducted with immunosuppressive cytokines in the TME, anti-tumor B cell subsets and IgG1 levels.

## Conclusion

It is increasingly appreciated that melanoma can develop intricate mechanisms to modulate the humoral immune response, and B cell immunity may also play a significant role in the efficacy and safety of CPI in melanoma. Taking all into consideration, it is highly conceivable that the promotion of anti-tumor B cell immunity with targeted therapies can also have clinical relevance in CPI response. Furthermore, immunomonitoring of humoral responses prior to, during, and following CPI in predicting irAEs should be explored. Given the complexity of tumor:immune cross-talk, utilizing B cells and antibodies as prognostic/predictive biomarkers in patient stratification and in immunomonitoring remains largely unexplored. Factoring in B cell immunity may harbor significant potential to overcome the two greatest challenges associated with CPI in melanoma: response and irAEs. Further studies in patients, *in vivo* and *ex vivo* models of immuno-oncology, alongside clinical testing, are required to fully comprehend and exploit likely multifaceted contributions.

## Author Contributions

ZNW, RJH, SC: Design and assembly of manuscript, design of illustrations. KH, HK, DT: Original draft writing. AnC, HJB, JC, AlC, RL, GO, RMH, MN, ST, LB: Planning, discussion and editing of the manuscript. JLG, AM-R, CH, JFS: Discussion, manuscript editing, clinical insights. SP, KEL, SNK: Manuscript design, project oversight. All authors contributed to the article and approved the submitted version.

## Funding

The authors acknowledge support by the Cancer Research UK King’s Health Partners Centre at King’s College London (C604/A25135); The Guy’s and St Thomas’s Foundation Trust Charity Melanoma Special Fund (573); CRUK/NIHR in England/DoH for Scotland, Wales, and Northern Ireland Experimental Cancer Medicine Centre (C10355/A15587); Breast Cancer Now (147; KCL-BCN-Q3); the Medical Research Council (MR/L023091/1); Cancer Research UK (C30122/A11527; C30122/A15774). The research was supported by the National Institute for Health Research (NIHR) Biomedical Research Centre (BRC) based at Guy’s and St Thomas’ NHS Foundation Trust and King’s College London (IS-BRC-1215-20006). The authors are solely responsible for study design, data collection, analysis, decision to publish, and preparation of the manuscript. The views expressed are those of the author(s) and not necessarily those of the NHS, the NIHR, or the Department of Health.

## Conflict of Interest

SNK and JFS are founders and shareholders of Epsilogen Ltd. HJB is now employed through a fund provided by Epsilogen Ltd.

The remaining authors declare that the research was conducted in the absence of any commercial or financial relationships that could be construed as a potential conflict of interest.
